# True good

**DOI:** 10.1186/1742-5581-1-1

**Published:** 2004-09-20

**Authors:** Charles J Greenberg

**Affiliations:** 1 Harvey Cushing/John Hay Whitney Medical Library, Yale University, 333 Cedar Street, PO Box 208014, New Haven, CT, 06850-8014, USA

"The open society, the unrestricted access to knowledge, the unplanned and uninhibited association of men for its furtherance – these are what may make a vast, complex, ever growing, ever changing, ever more specialized and expert technological world, nevertheless a world of human community [1]." –J. Robert Oppenheimer (1904–1967)

Biomedical scientific communities increasingly articulate concern about the unsustainable *status quo *of traditional publishing paradigms [2], feeding an ongoing debate and consideration of alternative publishing models [3]. Seizing an opportune moment to contribute to the ongoing assessment and refinement of scholarly publishing in biomedical education, research, and patient care, an energetic group of librarians and faculty researchers endeavour to launch *Biomedical Digital Libraries*.

While traditional biomedical library concern revolves around the role of collection stewardship and delivering published knowledge to students, scholars, researchers, and clinicians, information professionals in library or information center settings are also concerned with the cultivation of their own knowledge domains and evidence-based library practice [4,5]. Perhaps more than any other knowledge profession, librarians understand that their own opportunities for collaboration and barrier-free exchange of ideas and research results has dramatically altered and ultimately improved the provision of services and resource management in their professional environment. 

Open access scientific literature arrived in several pre-print guises over the previous two decades and in a recent contemporary commercial model with BioMed Central [6] (BMC). Separating itself from the pre-print unfiltered era, BMC provides biomedical researchers with peer review, retention of copyright, permanent redundant digital archiving in repositories such as PubMed Central (PMC) [7], and rapid global distribution of their ideas. Harnessing the innovations of the web, BMC also provides online submission, article history, and support for multiple languages. To sustain and expand an open access business model and maintain timely equitable global access, BMC publishing income derives from a combination of author fees, institutional memberships, and advertising. 

BMC now has 425 institutional members in 40 countries [8]. Researchers from member institutions have the right to publish an unlimited number of research articles in journals published by BMC without paying any article processing charges. Because of the BMC institutional membership, biomedical librarians and information professionals have unexpectedly been thrust into a position of advocacy for an alternative publishing model. More often than not, librarians are more aware than their faculty or researchers that open access does not mean the absence of peer review. In a web-based information age of unfiltered content and one-stop search engine shopping, both biomedical scientists and information professionals are justifiably concerned that the open access movement without peer review simply adds to the morass of unfiltered, unproven hyperbole. Attention to peer review provides credibility, and BMC even offers their journals the opportunity to publish review reports and preliminary drafts for each article as "publication history."

The decision to found a BMC journal for the world of organized biomedical information was both spontaneous and practical. At the core of the founding of *Biomedical Digital Libraries *is the conviction that open access will push biomedical librarianship forward in new and improved ways. *Biomedical Digital Libraries *provides a legitimate alternative to traditional specialty journals in the field, which have subscription fees and assumption of copyright by the publisher. This journal will stress peer-reviewed open access to research and practice in digital collection and services settings and will permit rapid and unimpeded dissemination of knowledge, only weeks after manuscript submission. Over 30 information professionals and biomedical scholars will be engaged as editorial staff to conduct blinded peer review reports for original research, as well as integrate commentaries, resource reviews, and debates into a forum for the discussion of unique considerations of biomedical information needs. These include both opportunities and constraints presented by health care settings, including collaborative initiatives with information technology and informatics partners.

We hope the advocates, philosophers, caretakers, and architects of biomedical library digital content take immediate advantage of rapid peer-review and publication, extensive BMC content promotion, permanent URL, redundant public archiving, and retention of copyright when they submit to *Biomedical Digital Libraries*. Beyond our immediate narrow spheres of digital library practice and service, the community of open knowledge has the immediate and timely potential to inspire, inform, and create value on a global scale through permanent, uninhibited access.

Some seek good in authority, others in scientific research, others in pleasure. Others, who are in fact nearer the truth, have considered it necessary that the universal good, which all men desire, should not consist in any of the particular things which can only be possessed by one man, and which, when shared, afflict their possessor more by the want of the part he has not, than they please him by the possession of what he has. They have learned that the true good should be such as all can possess at once, without diminution and without envy, and which no one can lose against his will [9].

– Blaise Pascal(1623–1662) 
